# Nanoparticle-Mediated RNAi Delivery System Targeting the *btCHS* and *btG6PI* Genes in *Bemisia tabaci*

**DOI:** 10.3390/insects17070737

**Published:** 2026-07-18

**Authors:** Jingjing Li, Yinghan Zhao, Chenchen Zhao, Zuyi Jia, Fengming Yan

**Affiliations:** College of Plant Protection, Henan Agricultural University, Zhengzhou 450046, China; jjli@henau.edu.cn (J.L.); zyh17647335365@163.com (Y.Z.); zhaochen@webmail.hzau.edu.cn (C.Z.); zuyi_jia@163.com (Z.J.)

**Keywords:** Chitin synthase, Glucose-6-phosphate isomerase, RNA interference, Nanoparticles, C_60_/alkylpolyglucoside, *Bemisia tabaci*

## Abstract

RNA interference (RNAi) technology has emerged as a promising approach to developing biopesticides. However, its effectiveness in the field depends largely on developing efficient and stable systems that can deliver double-stranded (ds)RNA to the intended target. In this study, we describe a novel dsRNA delivery system based on a nanomaterial (C_60_/ APG), and evaluated its RNAi efficiency systematically by targeting the chitin synthase (*btCHS*) and glucose-6-phosphate isomerase (*btG6PI*) genes of the agricultural pest *Bemisia tabaci*. The results showed that compared with dsRNA alone (30% and 40%), the ds*CHS* + Nano and ds*G6PI* + Nano groups showed significantly lower survival rates (≈10% and 25%) at 10 days post-treatment, as nanocarrier-delivered dsRNA was absorbed by tobacco plants and triggered RNAi against the target genes, inhibiting *B. tabaci* development. These findings confirm the role of nanocarriers in improving RNAi efficacy and support the development of sustainable nano-RNAi strategies for crop pest control.

## Simple Summary

RNA interference (RNAi) is an environmentally friendly method of pest control that works by silencing specific genes in insects; however, its effectiveness in real-world farming depends on its ability to deliver double-stranded (ds)RNA stably to the target pest. In this study, we developed a new delivery system using a nanomaterial called C_60_/alkylpolyglucoside (APG). We tested the effects of this system on the expression of two genes (chitin synthase and glucose-6-phosphate isomerase) essential for the growth and survival of the agricultural pest *Bemisia tabaci* (whitefly). The results show that the C_60_/APG nanomaterial enhanced dsRNA-mediated silencing efficiency, reducing *btCHS* and *btG6PI* expression in *B. tabaci* by 90%, compared with reductions of only 70% and 60%, respectively, when dsRNA was used alone. Overall, this study highlights the important role of nanocarriers in making RNAi more effective, and offers a practical foundation for developing sustainable nano-RNAi-based pest management strategies for crops.

## 1. Introduction

*Bemisia tabaci* (Gennadius) (Hemiptera: Aleyrodidae), an important agricultural pest [1,2,3], can transmit a variety of plant viruses that affect growth and development [4]. At present, it is considered one of the most destructive and invasive agricultural pests worldwide [1,3,5,6,7]. It has a wide range of host plants, including more than 900 species [1,6], and is distributed widely across more than 100 countries and regions (excluding Antarctica). Thus, *B. tabaci* is a globally significant agricultural pest [7,8,9].

RNA interference (RNAi) is a naturally occurring regulatory mechanism present in almost all eukaryotes [10,11]. Small interfering RNAs and microRNAs, typically ~21–24 nucleotides in length, are two classes of small single-stranded RNAs that play key roles in the RNAi pathway. These molecules recognize and degrade specific target RNAs to induce silencing of the corresponding genes [10,12,13]; however, traditional RNA-based pesticide delivery systems are prone to RNA degradation. Nanotechnology has been applied successfully within the field of biomedicine; therefore, combining nanotechnology with traditional agriculture offers promising opportunities, and provides a new paradigm for the advancement of modern agricultural science [14]. In the context of agriculture, pesticides are delivered mainly by nanocarriers, which carry exogenous insecticidal factors efficiently, enhance virulence, broaden the insecticidal spectrum, reduce the amount of insecticide required, prolong insecticide efficacy, and minimize environmental pollution. RNA pesticides based on nano delivery systems can improve the ability of double-stranded (ds)RNA to penetrate the pest body walls, and enhance the efficiency of RNAi [15,16]. In addition, they can be utilized as sprayable pesticides, which are more conducive to field operations; thus, these new insecticides have the prospect of broad application in the context of pest control. At the same time, nanocarriers protect dsRNA from environmental factors, improve the stability of dsRNA while it is being delivered into insects [17], and effectively penetrate the insect intestinal peritrophic membrane, cell membrane, and even body wall barriers, thereby increasing RNAi efficiency markedly and achieving more effective pest control [18,19,20]. The insect chitin synthesis pathway involves eight genes, including chitin synthase (*CHS*) and glucose-6-phosphate isomerase (*G6PI*): the most critical and final step of this pathway is catalyzed by CHS [21]. Trehalose metabolism can regulate the chitin synthesis pathway directly, leading to its disruption (particularly by decreasing chitin levels via reduced expression of the *CHS* gene). Furthermore, dysregulation of the CHS pathway can also disrupt insect molting and wing development, and even result in high mortality [22]. Nanotechnology-based RNAi as efficient dsRNA delivery methods are necessary for effective field pest control. Nanoparticles have been confirmed as an effective approach for delivering dsRNA and increasing efficiency [15].

RNAi has been widely explored for controlling *B. tabaci* [23,24,25,26]. Previous studies have demonstrated its feasibility through tissue-specific silencing [23], oral delivery of dsRNA targeting essential genes [24], and transgenic plant-mediated silencing. Several reviews have summarized key progress and challenges in this area [25,26,27,28,29]. However, dsRNA delivery efficiency remains a critical bottleneck, especially in hemipteran species like *B. tabaci*, which show relatively low RNAi sensitivity. Nanoparticle-based delivery systems offer a promising strategy to protect dsRNA from degradation and enhance cellular uptake. In this study, we employed a C_60_ nanocarrier to improve dsRNA delivery and evaluate its RNAi efficacy against *B. tabaci*.

## 2. Materials and Methods

### 2.1. B. tabaci, Tobacco Plants, and Growth Conditions

*B. tabaci* insects and *Nicotiana tabacum* (cv. Zhongyan 100) plants were reared in an artificial greenhouse at 25 ± 1 °C and a relative humidity (RH) of 70–80%, with a light:dark photoperiod of 16 h:8 h. The tobacco seedlings were cultured in cages (60 cm × 40 cm × 80 cm).

### 2.2. RNA Isolation and Cloning of Target Gene Fragments

Total RNA was extracted from tobacco plants and adults of *B. tabaci* using RNAiso Plus (Takara, Dalian, China) in accordance with the manufacturer’s instructions. Complementary DNA (cDNA) was synthesized using a PrimeScript™ FAST RT reagent Kit with gDNA Eraser (RR092A; Takara, Dalian, China), with 1 μg RNA used as a template. After PCR amplification, the target gene fragments (GFP = 521 bp; btG6PI = 541 bp (Gene ID: 109037018); btCHS = 557 bp (Gene ID: 109035684)) were inserted into plasmid pClone007 (Tsingke, TSV-007VS; Tsingke, Beijing, China), and then transformed into *Escherichia coli DH5α* (Tsingke, Beijing, China). Following sequence verification (by Sanger sequencing), the plasmids were used for subsequent experiments.

### 2.3. In Vitro Synthesis of dsRNA and dsRNA-Cy3

Fragments of GFP, *btG6PI,* and *btCHS* were PCR-amplified using specific primers containing the T7 promoter sequence and then purified. Next, dsRNA was synthesized using a T7 RNAi Transcription Kit (Vazyme, Nanjing, China), and dsRNA-Cy3 was synthesized using the HyperScribe T7 High Yield Cy3 RNA Labeling Kit (APExBIO, Houston, TX, USA). The concentration of dsRNA was determined using a nano-photometer (NanoDrop 1000; Thermo Fisher Scientific, Waltham, MA, USA). Following agarose gel electrophoresis, the size of the dsRNA bands, and whether they appeared as single bands, were determined. Samples were stored at −80 °C prior to subsequent experiments. Green fluorescent protein (GFP) was used as a negative control. The primers used for dsRNA synthesis are shown in Table S1.

### 2.4. Fusion of Nanomaterial C_60_ with dsRNA

First, we dissolved nanomaterial C_60_ in RNase-free H_2_O to concentrations of 3000 ng/μL and 1500 ng/μL. The fusion of C_60_ with dsRNA was done by mixing them at different mass ratios, and then alkyl glycoside APG-0816 was added to a final concentration of 1% for gel electrophoresis to check if they bind to each other. The fused dsRNA and C_60_ were also added to 1% SDS to separate dsRNA from the nanomaterial to confirm that dsRNA was fused with the nanomaterial by gel electrophoresis. The used fusion ratio was C_60_ to dsRNA = 3:1, with APG concentration at 1% for adult artificial diet and plant absorption experiments [20].

### 2.5. Nano-dsRNA-Mediated RNAi of the btG6PI and btCHS Genes in B. tabaci

To determine the RNAi efficacy of dsRNA when fed as part of an artificial diet, as well as in plant spraying and root soaking bioassays, naked dsRNA (500 ng/μL) and nano-dsRNA (a final concentration of 1500 ng/μL C_60_-dsRNA) were prepared in RNase-free water (dsGFP was used as a negative control). The artificial diet feeding apparatus comprised a centrifuge tube (5 cm in height and 2.2 cm in diameter), with the artificial diet (10% sucrose solution and 1% bovine serum albumin) contained within a double layer of Parafilm. About 30 *B. tabaci* insects were starved for 6 h and then allowed to feed on 200 μL of the artificial diet alone, or artificial diet containing naked dsRNA, nano-dsRNA, or dsGFP. The knockdown effects were then tested by qRT-PCR at 24 h post feeding.

### 2.6. Transfer of Nano-dsRNA into Tobacco Leaf Shoots

Next, Cy3-labeled nano-dsRNA was used to examine the efficiency with which dsRNA nanoparticles were transferred to the leaf cell. Fluorescence intensity was quantified by Image J software (Version 1.54p). All treatments and control experiments were performed in triplicate and all test groups were kept in incubators at 25 ± 1 °C and 70 ± 5% RH. After 24 h of incubation at 25 ± 1 °C and 70 ± 5% RH, the roots (8–10 cm in height) were soaked separately with 100 μL of C_60_-dsRNA-Cy3 (final concentration, 500 ng/μL dsRNA and 1500 ng/μL C_60_/APG), dsRNA-Cy3 (control), or RNase-free water (blank control). Slices of plant leaf were cut after 24 h. All samples were observed under a confocal microscope (Zeiss LSM 980, Carl Zeiss Microscopy GmbH, Jena, Germany) with ZEN software (version 3.4, Zeiss, Jena, Germany). The Cy3 signal was acquired using a Zeiss LSM 980 confocal microscope with an excitation wavelength of 555 nm and an emission detection at 570 nm. The images were captured using 15× objective lens.

### 2.7. Plant-Mediated RNAi Effects on B. tabaci

To assess whether dsRNA can be transported from the plant roots to the leaves, tobacco roots were soaked in 100 μL of C60/APG-dsRNA-cy3 (final concentration, 500 ng/μL of dsRNA and 1500 ng/μL C_60_/APG), dsRNA-cy3 (control), or RNase-free water (blank control). After 24 h, 30 adult *B. tabaci* were introduced into two micro-insect cages containing two pre-soaked tobacco leaves. The adults were removed after 24 h of oviposition, and 30 eggs were identified and their position marked after observation under a super-depth-of-field microscope (VHX-600E; Keyence, Itasca, IL, USA). The remaining eggs were removed gently. The positions of the 30 eggs were photographed and recorded, and egg hatching was monitored daily under the super-depth-of-field microscope (VHX-600E; Keyence, Itasca, IL, USA). After the eggs hatched into first-instar nymphs, the position of the nymphs was recorded and photographed. The body length and width (in μM) of the *B. tabaci* nymphs exposed to each treatment condition were observed and measured.

### 2.8. RT-qPCR

Surviving *B. tabaci* individuals collected 24 h after direct exposure were used for gene expression analysis. The expression of target genes in *B. tabaci* and the gene expression of *B. tabaci* target genes in tobacco plants were detected by RT-qPCR. Briefly, total RNA was extracted from 30 *B. tabaci* using RNAiso Plus (Takara, Dalian, China) and cDNA was synthesized using a PrimeScript RT Reagent Kit With gDNA Eraser (Perfect Real Time; Takara, Dalian, China). Each RT-qPCR reaction contained 5 μL of TB Green Premix Ex Taq II (Tli RNaseH Plus), 0.4 μL of forward and reverse primers (10 μmol/L), 2 μL of cDNA, 0.2 μL of ROX Dye II, and 2 μL of ddH_2_O (final reaction volume, 10 μL). The qPCR program was as follows: 95 °C for 1 min, followed by 40 cycles of 95 °C for 5 s and 60 °C for 40 s. The melting curve was determined after the reaction. All RT-qPCR assays were performed in triplicate. Relative gene expression levels were determined according to the 2^−ΔΔCT^ method.

### 2.9. Data and Statistical Analyses

All data were analyzed using SPSS Statistics 27.0. Graphs were created using GraphPad Prism 8.0. Data were analyzed using one-way ANOVA followed by least squares difference (LSD) multiple comparisons. Different letters indicate significant differences (*p* < 0.05). Differences among groups were examined using Student’s *t*-test, with significance defined as follows: * *p* < 0.05, ** *p* < 0.01, and *** *p* < 0.001, and ns, not significant. The survival curve results were analyzed using the log-rank (Mantel–Cox) test, with significance defined as * *p* < 0.05 and *** *p* < 0.001. 

## 3. Results

### 3.1. Membrane Feeding-Mediated Silencing of btCHS and btG6PI 

**Figure 1:**
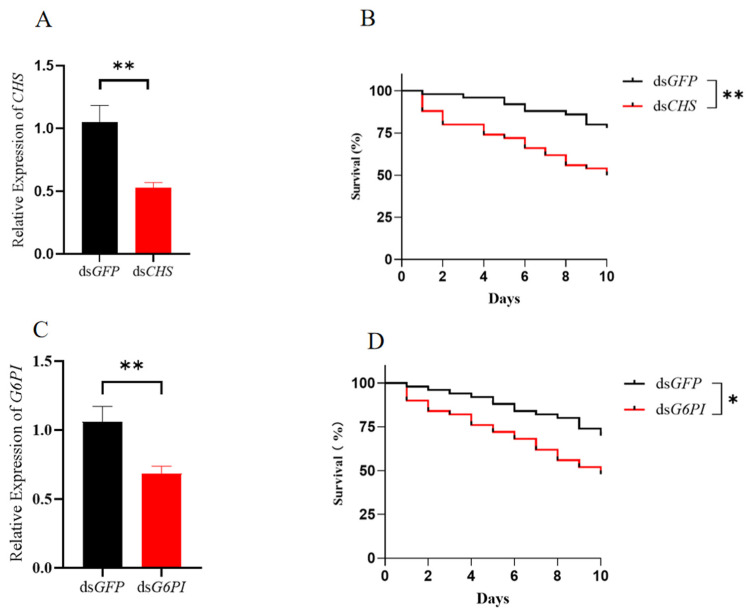
RNAi targeting *btCHS* and *btG6PI* expression, and survival rate of *B. tabaci*. (**A**,**B**) Effects of dsRNA on expression of the *btCHS* and *btG6PI* genes in *B. tabaci*. (**C**,**D**) Survival of *B. tabaci* versus expression levels of the *btCHS* and *btG6PI* genes. Statistical analysis was performed using one-way analysis of variance (ANOVA) followed by least squares difference (LSD) multiple comparison tests. The survival curve results were analyzed using the log-rank (Mantel–Cox) test (*n* ≈ 50 insects per experiment). Significant differences are indicated as * *p* < 0.05 and ** *p* < 0.01. The *p*-values are given at the bottom.

Inhibiting expression of the *btCHS* and *btG6PI* genes in pests through RNAi may disrupt their energy metabolism, leading to growth retardation, a decline in vitality, and even death. Therefore, to evaluate the efficacy of the target fragments, we first synthesized dsRNA in vitro, fed it artificially for 24 h to adults of *B. tabaci*, and then detected expression of the *btCHS* and *btG6PI* genes. The results showed that expression of both *btCHS* and *btG6PI* was inhibited significantly (Figure 1A,C), and that the mortality of *B. tabaci* increased significantly, over a 10-day period post feeding (Figure 1B,D).

### 3.2. Effect of Nano-Mediated dsRNA Administration on Expression of btCHS and btG6PI

**Figure 2:**
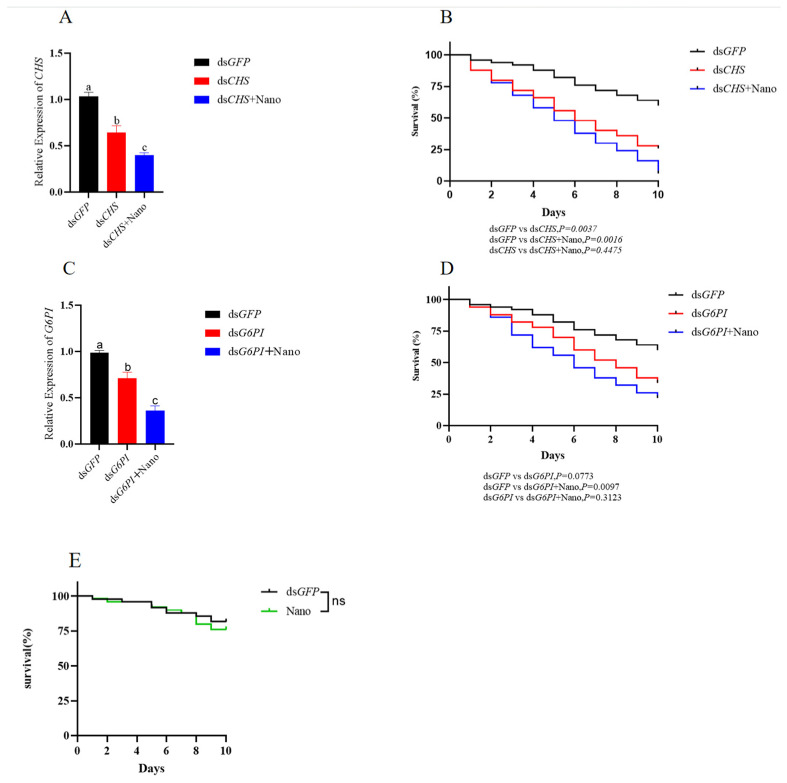
Gene expression and mortality of *B. tabaci* after feeding dsGFP, dsRNA, Nano or dsRNA+Nano. (**A**,**B**) Effects of artificial feeding of dsRNA and nano-dsRNA on expression of the *btCHS* and *btG6PI* genes in *B. tabaci*. (**C**,**D**) Effects of tobacco plant-absorbed dsRNA and nano-dsRNA on expression of the *btCHS* and *btG6PI* genes in *B. tabaci*. (**E**) Effects of feeding ds*GFP* and blank nanocarriers (1500 ng/μL) on the mortality of *B. tabaci*. Data were analyzed by one-way ANOVA followed by LSD multiple comparisons tests, and different letters indicate significant differences (*p* < 0.05). The survival curve results were analyzed using the log-rank (Mantel–Cox) test and the significance of the two survival curves (*n* ≈ 50 insects per experiment).“ns” indicates not significant. The *p*-values are shown under the survival graphs.

Firstly, as shown in Figure 2E, the C_60_/APG nanoparticles (1500 ng/μL) were non-toxic to *Bemisia tabaci*, as confirmed by feeding the whiteflies with the nanoparticles alone and dsGFP as controls. After the mixture of comprising 500 ng/μL dsRNA and 1500 ng/μL C_60_/APG was absorbed by the roots for 24 h, *B. tabaci* were transferred to the tobacco leaves and allowed to feed for a further 24 h. Expression of *CHS* and *G6PI* in *B. tabaci* was then detected. As shown in Figure 2A,C, expression of *btCHS* and *btG6PI* in *B. tabaci* decreased significantly (by 70% and 60%, respectively); furthermore, gene expression decreased more significantly (by 90%) in the presence of nanomaterial C60/APG, indicating that the nanomaterial enhances the silencing efficiency of dsRNA. According to the experimental results (Figure 2), treatments targeting the *btG6PI* gene (dsG6PI) and the *btCHS* gene (dsCHS) reduced the 10-day survival rate of *B. tabaci* significantly (by 60% and 65%, respectively). This effect was significantly better than that noted for the GFP control group. It is worth noting that the inhibitory effect of ds*btG6PI*+Nano and ds*btCHS*+Nano on the viability of *B. tabaci* was significantly stronger than that of the corresponding treatments without the nanomaterial. These results indicate that the C_60_/APG nanomaterial can effectively enhance the gene silencing efficiency of dsRNA, thereby increasing its lethal effect on *B. tabaci*. 

### 3.3. Carriage of dsRNA-Carrying Nanoparticles into Tobacco Plants via Application to the Roots

**Figure 3:**
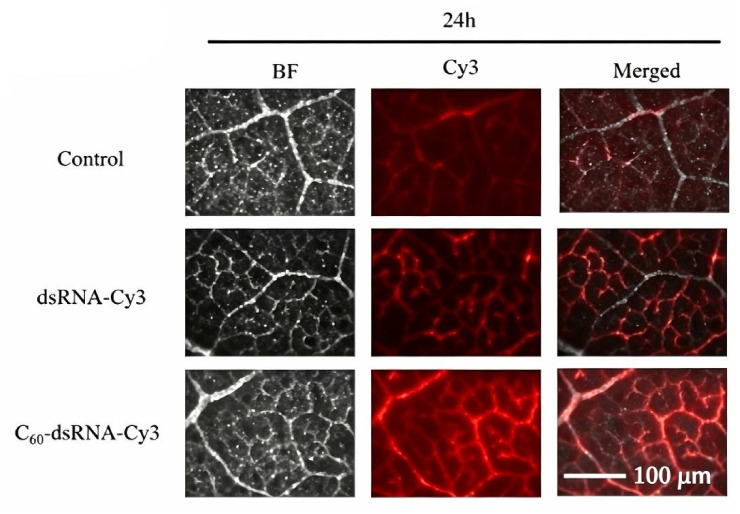
Distribution of nanoparticle-fused dsRNA within plant leaves. Images show leaves soaked for 24 h with either naked dsRNA or C_60_-dsRNA-Cy3. BF: bright field.

To explore whether the C_60_/APG nanomaterial promotes long-distance transport of dsRNA to tobacco leaves, the leaves were imaged under a fluorescence microscope after treatment with fluorescent-labeled dsRNA or C_60_/APG-dsRNA. Figure 3 shows that dsRNA was absorbed and transported to the leaves through tobacco stems, and that the intensity of the fluorescence signal was enhanced significantly after fusion of dsRNA with the C_60_/APG nanomaterial. These data indicate that the nanomaterial facilitates efficient transport of dsRNA into tobacco leaf tissues.

### 3.4. Application of RNAi Treatment to the Roots of Tobacco Plants Inhibits Growth of B. tabaci

**Figure 4:**
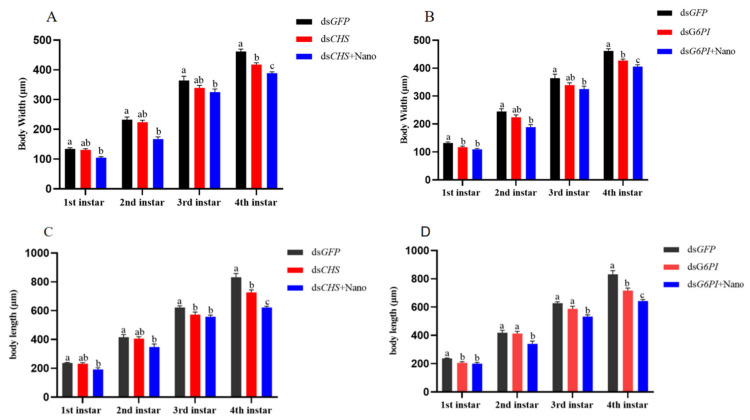
Comparison of *B. tabaci* body length and width after ingestion of tobacco-absorbed nanomaterial. (**A**,**B**) Effects of root application of ds*GFP*, ds*CHS*+Nano, and ds*G6PI*+Nano on the body width of *B. tabaci*. (**C**,**D**) Effects of root application of dsGFP, ds*CHS*+Nano, and ds*G6PI*+Nano on the body length of *B. tabaci*. Statistical analysis was performed using one-way ANOVA and LSD multiple comparisons tests, and different letters denote significant differences (*p* < 0.05).

Following RNAi-mediated knockdown of *btCHS*, both body width and body length were reduced across all four larval instars when compared with the control group (Figure 4A). In the control group, body width increased from 120 μm (1st instar) to 460 μm (4th instar), while body length increased from 240 μm to 840 μm. In the experimental group (Figure 4C), body width ranged from 115 μm to 420 μm, and body length from 220 μm to 820 μm, over the same developmental stages. The most pronounced effect was observed at the 3rd instar, where body width and body length were reduced by approximately 12.1% and 6.5%, respectively, suggesting a stage-specific sensitivity to *btCHS* silencing.

Silencing of *btG6PI* also resulted in reduced body width and body length relative to the control group (Figure 4B). In the control group, body width increased from 115 μm (1st instar) to 420 μm (4th instar), and body length from 220 μm to 820 μm. In the dsRNA-treated group (Figure 4D), body width ranged from 110 μm to 400 μm, and body length from 180 μm to 760 μm across the four instars. The greatest differences attributable to *btG6PI* silencing were again observed at the 3rd instar stage, with reductions of 9.0% in body width and 6.5% in body length. These findings indicate that silencing *btG6PI* impairs larval growth in both dimensions, with a relatively stronger effect on body width.

Taken together, these results indicate that RNAi targeting of the *btCHS* and *btG6PI* genes effectively impairs the growth and development of *B. tabaci*, and that the nanocarrier delivery system enhances the RNAi efficiency of dsRNA significantly, further aggravating developmental defects in body size. These morphological changes are consistent with the increased lethal effects demonstrated by the survival curves, suggesting that *btCHS* and *btG6PI* are key functional genes for the growth and development of *B. tabaci*, while also validating the synergistic role of nanocarriers in RNAi technology.

### 3.5. Effects of Root Application of dsRNA on Developmental Duration of B. tabaci

**Figure 5:**
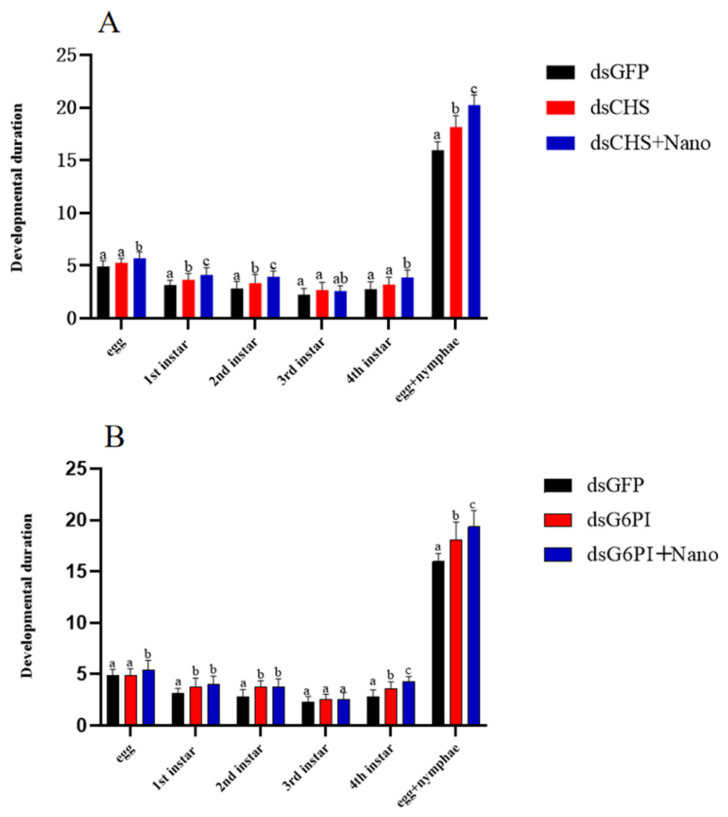
Comparison of the developmental duration of *B. tabaci* at different ages after treatment with dsGFP, dsRNA, or dsRNA+Nano. (**A**) Developmental duration of each instar of *B.tabaci* exposed to ds*CHS* and ds*CHS*+Nano. (**B**) Developmental duration of each instar of *B.tabaci* exposed to ds*G6PI* and ds*G6PI*+Nano. Statistical analysis was performed using one-way ANOVA and LSD multiple comparisons tests, and different letters denote significant differences (*p* < 0.05).

Figure 5A shows that application of ds*CHS* to tobacco plant roots resulted in significant differences in the developmental duration of the 1st and 2nd instar stages of *B. tabaci*, whereas there were no significant differences in the duration of the other instar stages. Following silencing of the *btCHS* gene, there was a significant increase in the developmental duration of the ds*CHS* and ds*CHS*+Nano treatment groups compared with the control group. As shown in Figure 5B, silencing of the *btG6PI* gene also prolonged the developmental duration of *B. tabaci*, with significant differences among the three groups at the fourth instar stage.

## 4. Discussion

RNAi technology is a new method of pest control based on the efficient delivery of stable dsRNA [17]. In this study, we used a C_60_/APG nanoparticle delivery system to successfully silence key genes in *B. tabaci*. The results show that this nano delivery system could expand application of RNAi to prevention and control of plant diseases, and provide the basis for future development of nanotechnology-based strategies for crop protection [13]. Nanocarriers not only improve the stability of dsRNA significantly, but also enhance its penetration ability, thereby increasing the efficiency of dsRNA uptake by *B. tabaci*. Taken together, our findings provide important evidence supporting the practical application of RNAi technology for crop management [30].

We found that in vitro-synthesized dsRNA can effectively interfere with the expression of the *btCHS* and *btG6PI* genes in *B. tabaci*, resulting in a significant impact on growth, development, and survival. Moreover, these effects were enhanced significantly when dsRNA was combined with nanomaterial C_60_/APG. Specifically, RNAi reduced the hatching rate and overall survival rate of *B. tabaci* significantly, with the body length and width of the treated *B. tabaci* being lower than those of the control group. ds*G6PI* combined with the nanomaterial also extended the developmental duration of *B. tabaci* at the 4th instar stage [31,32]. Thus, the data show that C_60_/APG nanoparticles effectively deliver dsRNA to the site of action and cause significant growth inhibition and lethal effects on *B. tabaci*, and that silencing of the *btCHS* and *btG6PI* genes confirms their key regulatory roles in the growth and development of *B. tabaci*. We acknowledge that prolonged nymphal development may theoretically extend feeding damage. However, this delay reflects a broader disruption to the insect life cycle, which also delays the emergence of the next generation. Consequently, while individual nymphs take longer to develop, overall population growth and cumulative damage from subsequent generations may be reduced. The trade-off between extended nymphal duration and suppressed population recruitment suggests that the net impact on plant damage is unlikely to be aggravated.

The protective function and delivery efficacy of nanomaterials have been confirmed by numerous studies. The formation of a complex between SPc and dsRNA can slow down the degradation of dsRNA by the haemolymph RNase of the citrus fruit fly (*Bactrocera dorsalis*) [33,34]. The construction and application of a star polycation nanocarrier-based delivery system for ds-MIRNAs have been demonstrated in *Arabidopsis* strains (Col) and a maize strain (ZD958) [35,36]. In addition, this SPc/dsRNA delivery platform significantly increases the mortality rate of cotton aphids (*Aphis gossypii*) and is capable of penetrating physical barriers such as eggshells and larval cuticles [37]. These findings corroborate the results of this study, collectively revealing the key role of nanocarriers in overcoming the bottlenecks in the application of RNAi technology [38,39].

The nanocarrier-mediated *btCHS* and *btG6PI* RNAi control system developed in this study, while achieving highly effective control of whiteflies, requires systematic assessment of its potential off-target ecological effects. As both *btCHS* and *btG6PI* are highly conserved functional genes essential for insect life processes, and their coding sequences exhibit a degree of homology within the class Insecta, there is theoretically a risk of off-target interference affecting natural enemies of the whitefly, non-target soil arthropods, and other beneficial organisms in agricultural fields. These organisms may be exposed through feeding on affected whiteflies or through contact with the dsRNA complexes present in plants or soil [40,41]. However, during the dsRNA fragment design phase of this study, NCBI BLAST (https://blast.ncbi.nlm.nih.gov/Blast.cgi, accessed date 15 July 2026) sequence alignment was used to prioritize highly variable intraspecific regions of the whitefly, effectively avoiding conserved cross-species domains [42]. Furthermore, the C_60_/APG nanocarriers used exhibit no biological toxicity at the experimental concentrations and serve solely to protect and deliver the dsRNA [43]. Additionally, as the RNAi effect is strictly sequence-dependent, exogenous dsRNA can degrade rapidly in the environment without the risk of residue or accumulation [44]. Therefore, this control system poses a low and manageable ecological risk to non-target organisms. Subsequent field applications may further refine the ecological safety assessment by incorporating safety testing of natural enemies and analysis of rhizosphere microorganisms.

To summarize, the combination of the C_60_/APG nanomaterial with RNAi technology may provide a solution to the poor stability and low delivery efficiency of dsRNA in field applications, and provide a new technical reserve for promoting the sustainable development of agriculture. On this basis, future studies should explore the best combinations of different nanomaterials and target genes to obtain an ideal field control effect.

## Data Availability

Data are available from the authors upon reasonable request.

## Supplementary Materials

The following supporting information can be downloaded at: https://www.mdpi.com/article/10.3390/insects17070737/s1, Table S1. List of primers used in the study.

## References

[1] Boykin L.M., Shatters R.G., Rosell R.C., McKenzie C.L., Bagnall R.A., De Barro P., Frohlich D.R. (2007). Global relationships of *Bemisia tabaci* (Hemiptera: Aleyrodidae) revealed using Bayesian analysis of mitochondrial COI DNA sequences. Mol. Phylogenetics Evol..

[2] Byrne D.N., Bellows T.S. (1991). Whitefly biology. Annu. Rev. Entomol..

[3] De Barro P.J., Liu S.S., Boykin L.M., Dinsdale A.B. (2011). *Bemisia tabaci*: A statement of species status. Annu. Rev. Entomol..

[4] Hernandez R.N., Isakeit T., Al Rwahnih M., Hernandez R., Alabi O.J. (2021). First report of Cucurbit chlorotic yellows virus infecting cantaloupe (*Cucumis melo* L.) in Texas. Plant Dis..

[5] Rosli M.A.F., Syed Jaafar S.N., Azizan K.A., Yaakop S., Aizat W.M. (2024). Omics approaches to unravel insecticide resistance mechanism in *Bemisia tabaci* (Gennadius) (Hemiptera: Aleyrodidae). PeerJ.

[6] Brown J.K., And D.R.F., Rosell R.C. (1995). The Sweetpotato or Silverleaf Whiteflies: Biotypes of *Bemisia tabaci* or a Species Complex. Annu. Rev. Entomol..

[7] Cuthbertson A.G.S., Vänninen I. (2015). The importance of maintaining protected zone status against *Bemisia tabaci*. Insects.

[8] Ghahari H., Abd-Rabou S., Zahradnik J., Ostovan H. (2009). Annotated catalogue of whiteflies (Hemiptera: Sternorrhyncha: Aleyrodidae) from Arasbaran, Northwestern Iran. J. Entomol. Nematol..

[9] Simmons A.M., Harrison H.F., Ling K.S. (2008). Forty-nine new host plant species for *Bemisia tabaci* (Hemiptera: Aleyrodidae). Entomol. Sci..

[10] Carthew R.W., Sontheimer E.J. (2009). Origins and Mechanisms of miRNAs and siRNAs. Cell.

[11] Jinek M., Doudna J.A. (2009). A three-dimensional view of the molecular machinery of RNA interference. Nature.

[12] Iwasaki Y.W., Siomi M.C., Siomi H. (2015). PIWI-Interacting RNA: Its biogenesis and functions. Annu. Rev. Biochem..

[13] Kumar A., Prasad R., Srivastava A.K., Singh B., Mishra R.K. (2020). Enhancing RNAi Efficiency to Decipher the Functional Response of Potential Genes in *Bemisia tabaci* AsiaII-1 (Gennadius) Through dsRNA Feeding Assays. Front. Physiol..

[14] Mathur S., Chaturvedi A., Ranjan R. (2025). Advances in RNAi-Based Nanoformulations: Revolutionizing Crop Protection and Stress Tolerance in Agriculture. Nanoscale Adv..

[15] Arjunan N., Thiruvengadam V., Sushil S.N. (2024). Nanoparticle-Mediated dsRNA Delivery for Precision Insect Pest Control: A Comprehensive Review. Mol. Biol. Rep..

[16] Qiao H., Chen J., Dong M., Shen J., Yan S. (2024). Nanocarrier-Based Eco-Friendly RNA Pesticides for Sustainable Management of Plant Pathogens and Pests. Nanomaterials.

[17] Yang W., Wang B., Lei G., Chen G., Liu D. (2022). Advances in nanocarriers to improve the stability of dsRNA in the environment. Front. Bioeng. Biotechnol..

[18] Liu J., He Q., Lin X., Smagghe G. (2025). Recent progress in nanoparticle-mediated RNA interference in insects: Unveiling new frontiers in pest control. J. Insect Physiol..

[19] Pugsley C.E., Isaac R.E., Warren N.J., Cayre O.J. (2021). Recent advances in engineered nanoparticles for RNAi-mediated crop protection against insect pests. Front. Agron..

[20] Wang X., Ji S., Bi S., Tang Y., Zhang G., Yan S., Wan F., Lu Z., Liu W. (2023). A promising approach to an environmentally friendly pest management solution: Nanocarrier-delivered dsRNA towards controlling the destructive invasive pest *Tuta absoluta*. Environ. Sci. Nano.

[21] Cohen E. (2001). Chitin synthesis and inhibition: A revisit. Pest Manag. Sci..

[22] Zhao C., Lou H., Liu Q., Pei S., Liao Q., Li Z. (2024). Efficient and transformation-free genome editing in pepper enabled by RNA virus-mediated delivery of CRISPR/Cas9. J. Integr. Plant Biol..

[23] Ghanim M., Czosnek H., Kontsedalov S. (2007). Tissue-specific gene silencing by RNA interference in the whitefly *Bemisia tabaci* (Gennadius). Insect Biochem. Mol. Biol..

[24] Upadhyay S.K., Chandrashekar K., Thakur N., Verma P.C., Borgio J.F., Singh P.K., Tuli R. (2011). RNA interference for the control of whiteflies (*Bemisia tabaci*) by oral route. J. Biosci..

[25] Grover S., Jindal V., Banta G., Taning C.N.T., Smagghe G., Christiaens O. (2019). Potential of RNA interference in the study and management of the whitefly, *Bemisia tabaci*. Arch. Insect Biochem. Physiol..

[26] Shelby E.A., Moss J.B., Andreason S.A., Simmons A.M., Moore A.J., Moore P.J. (2020). Debugging: Strategies and Considerations for Efficient RNAi-Mediated Control of the Whitefly *Bemisia tabaci*. Insects.

[27] Gong C., Yang Z., Hu Y., Wu Q., Wang S., Guo Z., Zhang Y. (2022). Silencing of the *BtTPS* genes by transgenic plant-mediated RNAi to control *Bemisia tabaci* MED. Pest Manag. Sci..

[28] Ibrahim A.B., Monteiro T.R., Cabral G.B., Aragão F.J.L. (2017). RNAi-mediated resistance to whitefly (*Bemisia tabaci*) in genetically engineered lettuce (*Lactuca sativa*). Transgenic Res..

[29] Luo Y., Chen Q., Luan J., Chung S.H., Van Eck J., Turgeon R., Douglas A.E. (2017). Towards an understanding of the molecular basis of effective RNAi against a global insect pest, the whitefly *Bemisia tabaci*. Insect Biochem. Mol. Biol..

[30] Jain R.G., Robinson K.E., Mitter N. (2019). RNAi-Mediated Management of Whitefly *Bemisia tabaci* by Oral Delivery of Double-stranded RNAs. Proceedings.

[31] Kong D., Gu H., Gao Y., Hou Y., Li J. (2025). Nanomaterial-Mediated RNAi Targeting Chitin Metabolism Genes in MEAM1 Cryptic Species of *Bemisia tabaci* (Hemiptera: Aleyrodidae). Insects.

[32] Ren Q., Zhang N., Liu Y., Li S., Zhang J., Wang Y., El Wakil A., Moussian B., Zhang J. (2025). PEI-SWNT improves RNAi efficiency in *Locusta migratoria* via dsRNA injection delivery system. Pestic. Biochem. Physiol..

[33] Dhandapani R.K., Gurusamy D., Palli S.R. (2021). Development of Catechin, Poly-l-lysine, and Double-Stranded RNA Nanoparticles. ACS Appl. Bio Mater..

[34] Qiao H., Jiang Q.H., Zhao J., Xiao L., Zhu-Salzman K., Xu D., Xu G., Shen J., Gu A., Hao D. (2025). Nano-delivery platform with strong protection and efficient delivery: Preparation of self-assembled RNA pesticide with dual RNAi targets against *Apolygus lucorum*. J. Nanobiotechnology.

[35] Wei Z.H., Zhao P., Ning X.Y., Xie Y.Q., Li Z., Liu X.X. (2024). Nanomaterial-Encapsulated dsRNA-Targeting Chitin Pathway—A Potential Efficient and Eco-Friendly Strategy against Cotton Aphid, *Aphis gossypii* (Hemiptera: Aphididae). J. Agric. Food Chem..

[36] Ma Z., Zheng Y., Chao Z., Chen H., Zhang Y., Yin M., Shen J., Yan S. (2022). Visualization of the process of a nanocarrier-mediated gene delivery: Stabilization, endocytosis and endosomal escape of genes for intracellular spreading. J. Nanobiotechnology.

[37] Zhao L., Yang M., Shen Q., Liu X., Shi Z., Wang S., Tang B. (2016). Functional characterization of three trehalase genes regulating the chitin metabolism pathway in rice brown planthopper using RNA interference. Sci. Rep..

[38] Fire A., Xu S., Montgomery M.K., Kostas S.A., Driver S.E., Mello C.C. (1998). Potent and specific genetic interference by double-stranded RNA in *Caenorhabditis elegans*. Nature.

[39] Bernstein E., Caudy A., Hammond S.M., Hannon G.J. (2001). Role for a bidentate ribonuclease in the initiation step of RNA interference. Nature.

[40] Joga M., Zotti M., Smagghe G. (2016). RNAi Efficiency, Systemic Properties, and Novel Delivery Methods for Pest Insect Control: What We Know So Far. Front. Physiol..

[41] Bachman P., Fischer J., Song Z., Urbanczyk-Wochniak E., Watson G. (2020). Environmental Fate and Dissipation of Applied dsRNA in Soil, Aquatic Systems, and Plants. Front. Plant Sci..

[42] Jain R.G., Fletcher S.J., Manzie N., Robinson K.E., Li P., Lu E., Brosnan C.A., Xu Z.P., Mitter N. (2022). Foliar application of clay-delivered RNA interference for whitefly control. Nat. Plants.

[43] Yan S., Ren B.Y., Shen J. (2021). Nanoparticle-mediated double-stranded RNA delivery system: A promising approach for sustainable pest management. Insect Sci..

[44] Zarrabian M., Sherif S.M., Shukla M.R. (2025). Stability of naked and encapsulated dsRNA in water and on plant surfaces: Key data to inform environmental safety and toxicity assessments. Ecotoxicol. Environ. Saf..

