# Supplementary material for: Nanoparticle-Mediated RNAi Delivery System Targeting the btCHS and btG6PI Genes in Bemisia tabaci
Source: Insects. 2026 Jul 18;17(7):737. doi: 10.3390/insects17070737 (PMC13411593; doi:10.3390/insects17070737)
Supplement: Supplementary file 1 [file insects-17-00737-s001.zip › insects-4315721-supplementary.pdf]

Table S1. List of primers used in the study

Primer	Sequence（5′→3′）	Amplicon size (bp)
GFP-F	GGATCCTAATACGACTCACT	276
GFP-R	TGGATCCTAATACGACTCAC	
btCHS-F	GCAAGTATACCTGCAGAAGA	612
btCHS-R	GAGCATAGAACAGAAGAAGC	
btG6PI-F	GCAGCTATTCAACGAAGACC	430
btG6PI-R	TTCTGTCACCATGTATGGGC	
GFP-T7-F	GATCACTAATACGACTCACTATAGGGGGA TCCTAATACGACTCACT	276
GFP-T7-R	GATCACTAATACGACTCACTATAGGGTGG ATCCTAATACGACTCAC	
btCHS-T7-F	GATCACTAATACGACTCACTATAGGGGCA AGTATACCTGCAGAAGA	612
btCHS-T7-R	GATCACTAATACGACTCACTATAGGGGAG CATAGAACAGAAGAAGC	
btG6PI-T7-F	GATCACTAATACGACTCACTATAGGGGCA GCTATTCAACGAAGACC	430
btG6PI-T7-R	GATCACTAATACGACTCACTATAGGGTTCT GTCACCATGTATGGGC	
btCHS-qRT-F	GCGGACCATTGGCACTCTTG	135
btCHS-qRT-R	GCTTCCCGAATCTCTCGCCG	
btG6PI-qRT-F	GTATGTAAACAGAAGTGGTC	130
btG6PI-qRT-R	ATCAGCGGGGATAAGTCTAG	
Action-F	TCTTCCAGCCATCCTTCTTG	174
Action-R	CGGTGATTTCCTTCTGCATT	
